# Anti-Ro/SSA antibodies in adult arrhythmias: pathogenesis, clinical implications, and therapeutic strategies

**DOI:** 10.3389/fimmu.2025.1561061

**Published:** 2025-05-05

**Authors:** Ruotong Cao, Huasheng Lv, Baopeng Tang, Yanmei Lu

**Affiliations:** ^1^ Department of Pacing and Electrophysiology, The First Affiliated Hospital of Xinjiang Medical University, Urumqi, China; ^2^ Department of Cardiac Electrophysiology and Remodeling, The First Affiliated Hospital of Xinjiang Medical University, Urumqi, China

**Keywords:** anti-ro/ssa antibodies, arrhythmia, adults, autoimmunity, immunomodulatory therapy

## Abstract

Anti-Ro/SSA antibodies, prevalent autoantibodies in connective tissue diseases, have well-established roles in fetal arrhythmias but their significance in adult arrhythmias remains underrecognized. Recent evidence highlights that anti-Ro/SSA antibodies may induce adult arrhythmias by disrupting cardiac ion channel function, particularly through interactions with calcium and potassium channels, leading to electrophysiological disturbances including QT prolongation, atrioventricular block, and increased susceptibility to sudden cardiac death. Additionally, these antibodies can initiate inflammatory cascades, further contributing to myocardial fibrosis and conduction abnormalities. Despite the growing clinical relevance, detection of anti-Ro/SSA antibodies in unexplained arrhythmias is not routinely performed, limiting early recognition and intervention. Therapeutic strategies, currently based primarily on immunomodulatory therapies, show promise yet lack definitive evidence from randomized controlled trials. This review systematically summarizes recent advances regarding the pathogenic mechanisms, clinical implications, and therapeutic strategies for anti-Ro/SSA antibody-associated adult arrhythmias, aiming to enhance awareness, diagnostic precision, and management of this increasingly recognized clinical entity.

## 1 Introduction

Cardiac arrhythmias, encompassing a spectrum of cardiovascular disorders ranging from mild palpitations to cardiac arrest, significantly impair patients’ quality of life and prognosis. In recent years, research has increasingly highlighted the pivotal role of autoimmunity in arrhythmogenesis. Among the various autoantibodies associated with autoimmune diseases, the potential role of anti-Ro/SSA antibodies in adult arrhythmias has garnered considerable attention (1–5). The link between anti-Ro/SSA antibodies and cardiac dysfunction was first established in congenital heart block (CHB), where maternal autoantibodies target fetal L- and T-type calcium channels, leading to conduction abnormalities and sinus bradycardia (6, 7). Clinical studies further show that maternal anti-Ro/SSA antibody levels ≥50 U/ml increase the risk of fetal CHB (OR:7.8, P<0.0001), with a 5% occurrence rate above this threshold, while no cases are observed below it, highlighting the need for prenatal monitoring in antibody-positive pregnancies (8, 9). Intriguingly, similar electrophysiological disturbances such as AV block and QT prolongation have been observed in anti-Ro/SSA-positive adults (10), suggesting a potential continuum of antibody-mediated pathology beyond fetal life. However, the mechanisms in adults may involve additional factors, including coexisting autoimmunity or age-related changes. This paper aims to systematically review the fundamental aspects of anti-Ro/SSA antibodies, explore their mechanistic roles and clinical significance in adult arrhythmias, and provide evidence-based insights for clinical treatment based on the latest research findings.

## 2 Anti-Ro/SSA antibodies

Anti-Ro/SSA antibodies are polyclonal IgG antibodies that primarily target the Ro52 and Ro60 antigen proteins. These antibodies were first identified in patients with systemic lupus erythematosus (SLE) (11) and Sjögren’s syndrome (SS) (12), and are among the most commonly recognized autoantibodies. Anti-Ro/SSA antibodies are closely associated with various connective tissue diseases (CTD) but are also detectable among apparently healthy individuals (13). In patients with SS, the prevalence of anti-Ro/SSA antibodies is as high as 70% to 95%, with anti-Ro52 antibodies showing greater specificity. In patients with SLE, positivity rates range from 30% to 50%. Furthermore, anti-Ro/SSA antibodies are detected in up to 60% of patients with subacute cutaneous lupus erythematosus (14–16). In contrast, positivity rates in healthy populations range from 0.5% to 3% (17–19), with an elevated prevalence of 8.3% reported among American veterans aged 50 to 60 years (1).

The Ro52 and Ro60 proteins, targeted by anti-Ro/SSA antibodies, are localized in the cytoplasm and nucleus, respectively. Ro52 is an interferon-induced protein with E3 ubiquitin ligase activity, while Ro60 is primarily involved in RNA polymerase III synthesis and rRNA degradation. These antigens play a significant role in disease pathogenesis by triggering autoantibody production and subsequent immune system activation (20, 21). Anti-Ro/SSA antibodies are strongly associated with CHB in neonatal lupus syndrome. Eftekhari et al. (22) first elucidated the molecular mechanism, demonstrating that anti-Ro52 antibodies cross-react with the second extracellular loop of cardiac 5-HT4 serotonin receptors, disrupting calcium channel activity and leading to fetal conduction abnormalities. Xiao et al. (6) further found that IgG from anti-SSA/Ro-positive mothers selectively inhibits L- and T-type calcium channel currents in Xenopus oocytes. Immunoblotting confirmed that the IgG cross-reacts with the pore-forming α1C subunit of L-type calcium channels (LTCC), with no significant effect on sodium (INa-hH1) or potassium (IKs-minK+KvLQT1) channels, indicating high selectivity for calcium channels. Additionally, Xiao’s rabbit model demonstrated that anti-Ro52 antibodies downregulate LTCC, providing new experimental evidence for CHB pathogenesis (23). Based on these findings, researchers propose a pathological model: Maternal anti-SSA/Ro antibodies cross the placenta, directly inhibiting L- and T-type calcium channels in fetal cardiomyocytes, leading to conduction defects and pacemaker dysfunction. Prolonged channel suppression triggers cellular stress, apoptosis, and ultimately inflammation, fibrosis, and permanent structural damage. Karnabi et al. (24) provided direct support for this hypothesis: LTCC-overexpressing mice resisted anti-Ro/La antibody-induced bradycardia and AV block, while LTCC-knockout mice exhibited severe arrhythmias. Peter M. Izmirly et al. (25) further detailed how maternal anti-SSA/Ro antibodies target fetal cardiac structures (e.g., Ro52-p200 epitope and LTCC), triggering abnormal immune responses in apoptotic cardiomyocytes and activating profibrotic cascades (uPA/TGF-β/ET-1), ultimately causing conduction block and myocardial injury. Szendrey et al. (26) identified the S5-pore linker of hERG channels as a key target for anti-Ro52 antibodies. These antibodies do not directly block hERG/Ikr current but promote hERG channel internalization and degradation, chronically reducing channel expression and current amplitude, leading to acquired long QT syndrome (LQTS). While their impact on fetal arrhythmias has been extensively studied, their role in adults remains relatively underexplored. However, emerging evidence suggests that anti-Ro/SSA antibodies may also contribute to arrhythmias in adults (27, 28). The detection of anti-Ro/SSA antibodies primarily relies on three methods: fluoroenzyme immunoassay (FEIA), immunoblotting (iWB), and line immunoassays. Among these, iWB demonstrates greater sensitivity for detecting the anti-Ro/SSA-52kD subtype (5). Studies have shown that combining FEIA with iWB significantly increases the detection rate of antibody positivity in patients with QTc(Corrected QT interval) prolongation (from 75% to 87.5%). This suggests that a combined testing approach enhances the sensitivity of detecting anti-Ro/SSA antibodies, particularly the 52kD subtype, and provides more reliable evidence for clinical diagnosis (10, 29).

## 3 Anti-Ro/SSA antibodies and adult arrhythmias

Anti-Ro/SSA antibodies are common autoantibodies, with a positivity rate up to ≈8% in the apparently healthy general population (1, 14–16). In fact, most antibody-positive individuals remain asymptomatic for autoimmune diseases. In recent years, studies have found that anti-Ro/SSA antibodies may be closely associated with the onset of arrhythmias in some patients with unexplained cardiac conditions (15). A large retrospective study by Villuendas et al. (30) revealed that among patients with isolated atrioventricular block (AVB) of unknown etiology who received pacemakers between 1987 and 2012, approximately 10.5% were positive for anti-Ro/SSA antibodies. This finding indicates a potential role of anti-Ro/SSA antibodies in the pathogenesis of isolated AVB in adults. The cross-sectional study by Gamazo-Herrero et al (31) confirmed a significant association between anti-Ro/SSA antibodies and increased risk of arrhythmias in adult patients with systemic autoimmune diseases (SADs). Notably, the incidence of QT interval prolongation reached 17.9% (RR=4.25) in anti-Ro/SSA-positive patients, with a markedly higher risk observed in strongly positive cases (27.8% vs. 10.5%).

**Figure 1:**
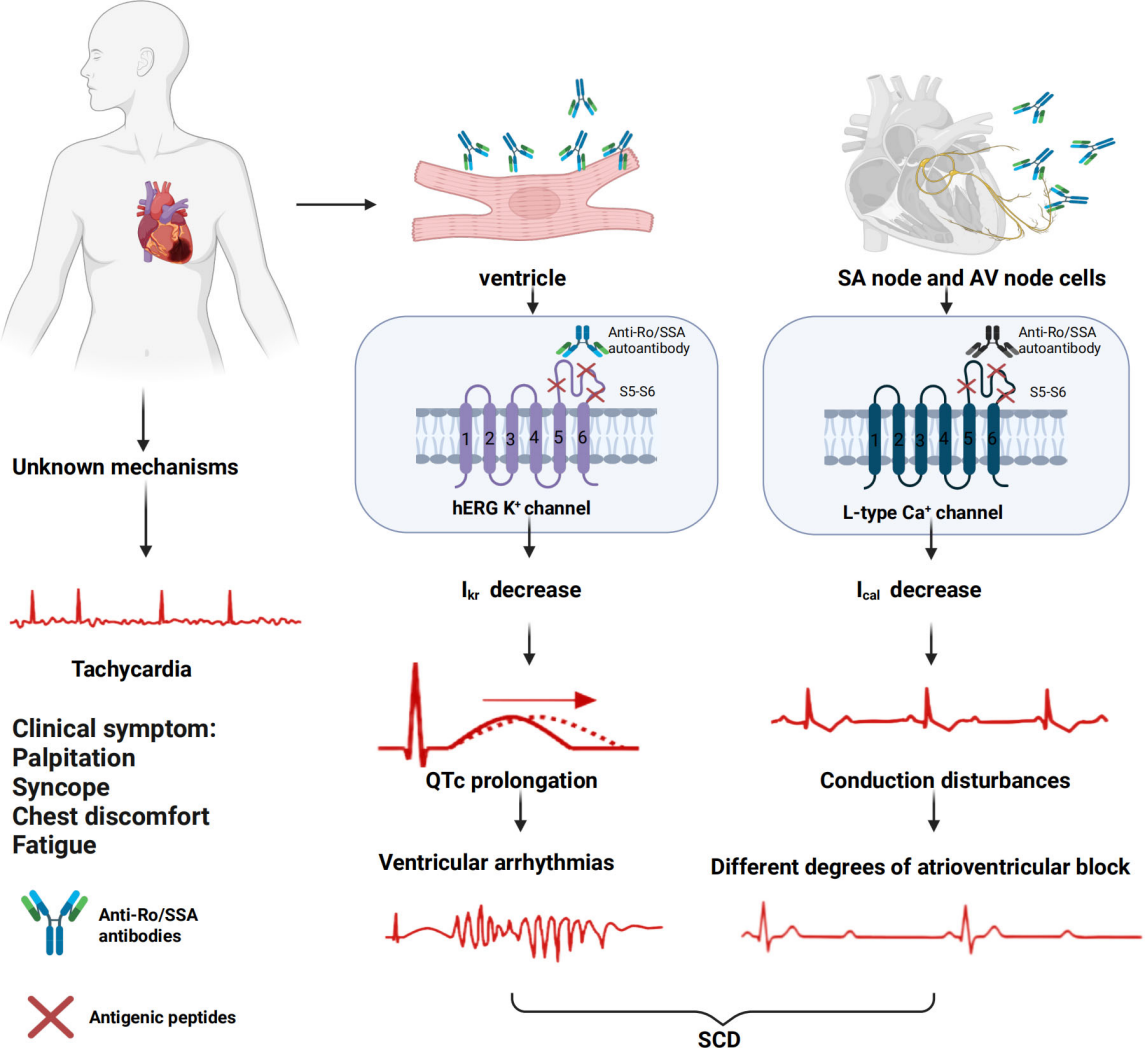
Mechanisms of anti-Ro/SSA antibody-associated arrhythmias. Anti-Ro/SSA antibodies interfere with cardiac ion channel function, primarily by binding to hERG potassium channels and L-type calcium channels, leading to a reduction in IKr and ICaL currents. These disruptions result in QTc prolongation, conduction abnormalities, ventricular arrhythmias, and atrioventricular block, significantly elevating the risk of sudden cardiac death (SCD). Tachycardia may also occur, although the underlying mechanisms are less clearly understood. Clinically, these effects present as symptoms such as palpitations, syncope, chest discomfort, fatigue, and potentially life-threatening arrhythmias.

Although traditional views hold that adult hearts lack immune targets for antibody action, growing evidence suggests that anti-Ro/SSA antibodies may induce arrhythmias by interfering with cardiac ion channel function (15). Studies have identified the disruption of calcium and potassium channels by anti-Ro/SSA antibodies as one of the key mechanisms underlying arrhythmogenesis (32). **Figure 1** provides a visual summary of these mechanisms, illustrating how anti-Ro/SSA antibodies disrupt ion channel function, particularly by binding to hERG potassium channels and LTCC. These interactions result in reduced IKr and ICaL currents, contributing to QTc prolongation, conduction disturbances, and increased susceptibility to arrhythmias.

**Figure 2:**
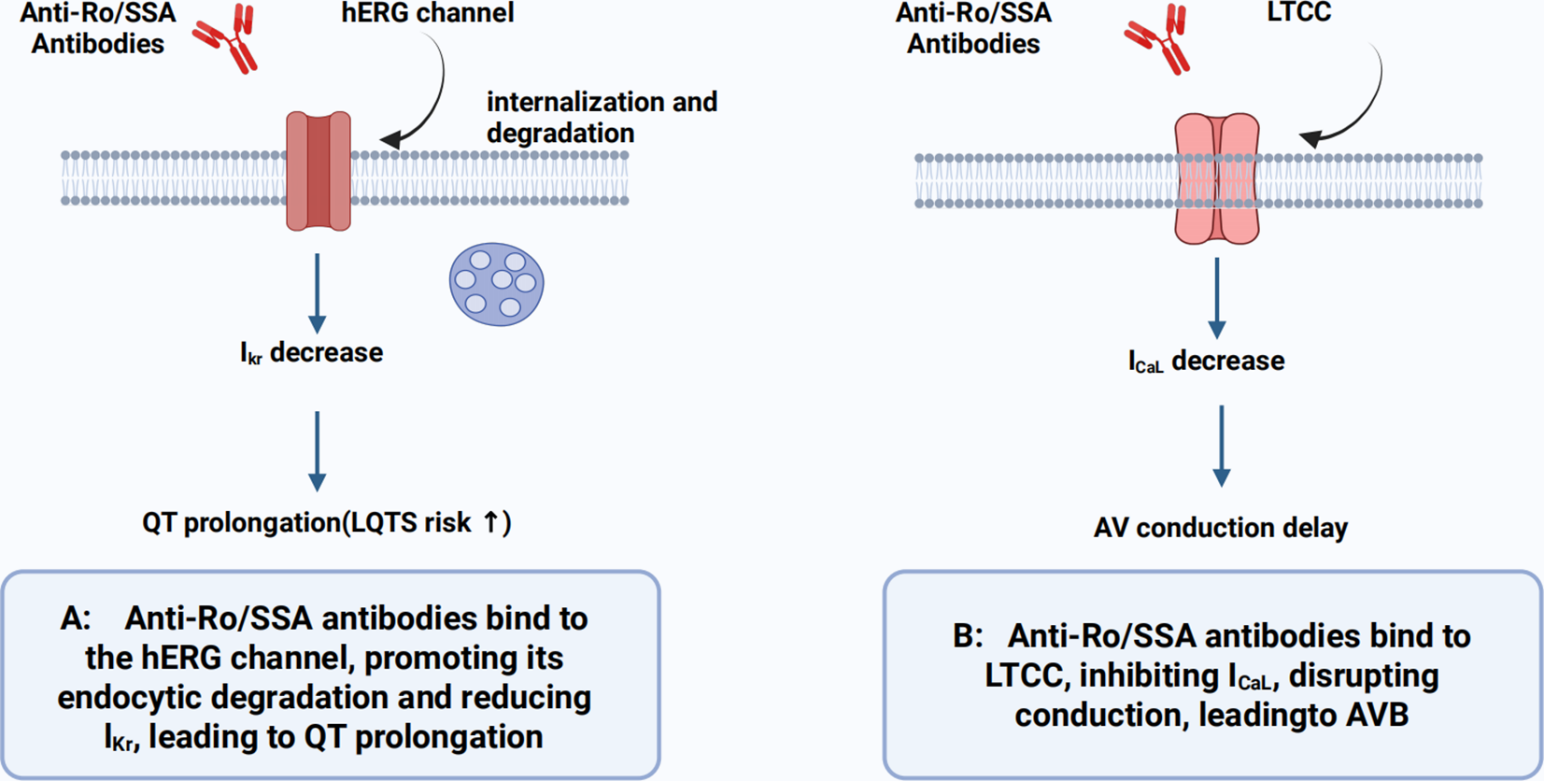
Autoantibody interactions with cardiac ion channels. **(A)** Anti-Ro/SSA antibodies bind to hERG channels, promoting internalization and reducing IKr, leading to QT prolongation. **(B)** Binding to Cav1.2 (L-type calcium channels) inhibits ICaL, disrupting atrioventricular conduction and contributing to AV block.

In adult cardiomyocytes, the expression levels of LTCC are significantly higher than in fetal cells, with corresponding current densities and mRNA levels being 2 to 4 times and 4 to 9 times greater, respectively (33). Additionally, adult cardiomyocytes exhibit stronger sarcoplasmic reticulum calcium storage capacity and lower apoptosis rates. These physiological differences may account for the lower incidence of irreversible AVB in adults compared to fetuses (34, 35). In anti-Ro/SSA antibody-positive patients, the modulation of ion channel function by inflammatory cytokines and the promotion of myocardial fibrosis might further and significantly increase the risk of arrhythmias. These mechanisms involve direct antibody-channel interactions and inflammatory signaling cascades. **Figure 2** illustrates these pathogenic pathways (36).

### 3.1 QT prolongation and sudden cardiac death

The QTc reflects the duration of ventricular action potentials, determined by the transmembrane flow of sodium and calcium ion influx and potassium ion efflux during repolarization. The most frequent electrocardiographic abnormality in anti-Ro/SSA antibody-positive adults is QTc prolongation, which may lead to LQTS, increasing the risk of syncope, cardiac arrest, and sudden cardiac death (SCD) (37, 38). LQTS is characterized by a heart rate-corrected QTc prolongation, with current guidelines defining abnormal QTc values as exceeding 470 milliseconds for males and 480 milliseconds for females (99th percentile thresholds) (39). Studies suggest that immunomodulatory therapy may be effective in managing QT interval prolongation (34).

In 2007, the first case of anti-KCNH2/hERG (human ether-a-go-go-related gene) antibody-induced LQTS was reported (13). Studies demonstrated that serum and purified IgG antibodies from an otherwise healthy anti-Ro/SSA-positive woman with severe QTc prolongation and torsades de pointes (TdP) could bind directly to the hERG K^+^ channel and inhibit the Rapidly Activated Delayed Rectifier Potassium Current (IKr). This report provides initial evidence that cross-reactivity between anti-Ro/SSA antibodies and the KCNH2 channel may underlie autoimmune LQTS. Such cross-reactivity may impair cardiac potassium channel function, disrupt the cardiac repolarization process, and lead to QTc prolongation and TdP, posing life-threatening risks (13). This study highlights the potential link between autoimmunity and cardiac electrophysiological abnormalities. Subsequent studies confirmed and expanded these findings. Anti-Ro/SSA antibodies bind to the S5-S6 region of the hERG/KCNH2 channel, a region that exhibits significant sequence homology with the Ro52 antigen (3). Research by John Szendrey et al. (26) further demonstrated that anti-Ro/SSA antibodies target the S5-pore linker of hERG, promoting its endocytic degradation, reducing hERG protein expression, and inhibiting potassium ion currents, thereby prolonging repolarization. Additionally, the heterogeneity of ventricular wall cardiomyocytes increases ventricular repolarization dispersion. QTc prolongation and IKr inhibition also enhance the likelihood of sodium and calcium channel reactivation, generating single or repeated membrane potential oscillations and increasing the risk of malignant arrhythmias such as TdP (40). Furthermore, anti-Ro/SSA antibodies can reduce ventricular repolarization reserve, significantly elevating the risk of ventricular arrhythmias. This phenomenon aligns with the multiple-hit theory, which posits that the combined effects of multiple factors may contribute to the onset of arrhythmias (41).

Among patients with TdP, approximately 60% are positive for anti-Ro/SSA antibodies (42), further confirming the strong association between anti-Ro/SSA antibodies and malignant arrhythmias. The risk of TdP increases progressively with QTc prolongation. Studies have shown that for every 10 ms increase in QTc, the risk of TdP rises by approximately 5% to 7%, and the risk becomes particularly significant when QTc exceeds 500 ms (39). Additionally, multiple studies indicate that even in the absence of a clear history of autoimmune diseases, anti-Ro/SSA antibodies can still elevate the risk of ventricular arrhythmias and sudden cardiac arrest (1, 37). In summary, anti-Ro/SSA-52kD antibodies not only impair channel function through direct blockade but may also exert more sustained effects by altering the cell surface expression of hERG channels and reducing ventricular repolarization reserve.

In addition, inflammatory cytokines such as IL-6 can phosphorylate the serine residue at position 1829 of the Cav1.2 channel, enhancing its function and leading to an increase in L-type calcium current (ICaL) (43). Similarly, interleukin-1 (IL-1) exerts consistent effects by prolonging the action potential duration (APD), reducing Ito through the lipoxygenase pathway, and increasing ICaL, thereby contributing to QTc prolongation and the development of LQTS. Studies have found elevated levels of IL-1β in patients with CTD who exhibit anti-Ro/SSA antibodies and prolonged QTc intervals (44). This further underscores the potentially significant role of inflammatory cytokines in the pro-arrhythmic effects of anti-Ro/SSA antibodies.

Recent studies suggest that anti-KCNQ1 antibodies may interact with the slowly activated delayed rectifier potassium channel (IKs), increasing IKs current during repolarization and thereby shortening the QT interval. This finding provides new insights into the treatment of immune-mediated LQTS (45). The IKs current is mediated by a heterotetrameric potassium channel composed of the Kv7.1 protein encoded by the KCNQ1 gene and the KCNE1 protein (also known as minK) encoded by the KCNE1 gene. This channel plays a critical role in the repolarization phase of the cardiac action potential, particularly during the late phase of ventricular cardiomyocyte repolarization, where it contributes to shortening the action potential duration.

In recent years, the impact of immune factors, particularly anti-Ro/SSA antibodies, on cardiac electrophysiological function and their role in arrhythmias has emerged as a significant area of research. Anti-Ro/SSA antibodies may contribute to immune-mediated LQTS by impairing hERG-K+ channel function and inhibiting IKr, ultimately leading toTdP and increasing the risk of SCD (46, 47). Recent advances have further elucidated the pathogenic role of autoantibodies in cardiac electrophysiological disorders. In a study involving 50 Brugada syndrome (BrS) patients and 50 healthy controls, Tarantino et al. (48) demonstrated that anti-Nav1.5 antibodies were detectable in 90% of BrS patients (vs. 6% in controls). Mechanistically, these antibodies suppressed sodium channel current density (~40% reduction in INa), thereby inducing characteristic ST-segment elevation in right precordial leads and ventricular arrhythmias. Critically, animal model experiments confirmed that passive transfer of plasma from BrS patients recapitulated Brugada-like electrocardiographic phenotypes and heightened the risk of malignant arrhythmias. These findings suggest that systematic screening for cardiac ion channel autoantibodies may redefine diagnostic classification in “idiopathic” arrhythmias while guiding individualized immunomodulatory therapies.

Lazzerini et al. (49) compared 26 anti-SSA/Ro-positive patients with 20 anti-SSA/Ro-negative patients and found that the incidence of complex ventricular arrhythmias (Lown grade 2 to 5) was significantly higher in the anti-SSA/Ro-positive group (50% vs. 10%), thereby elevating the risk of SCD. A correlation may also exists between anti-Ro/SSA antibody positivity and SCD, particularly in cases of sudden death without a clear underlying cause, where anti-Ro/SSA antibodies may serve as a potential pathogenic factor (36, 50). A recent observational cross-sectional study identified autoantibodies targeting the pore region of L-type voltage-gated calcium channels as potential biomarkers for idiopathic cardiac arrest (P=0.002; false discovery rate, 0.007; classification accuracy ≥0.83) (51).Of note, it has been demonstrated that this peptide sequence is the same recognized by anti-Ro/SSA antibodies (5, 52). Although coronary artery disease and heart failure are common causes of SCD, up to 15% of SCD cases show no significant structural cardiac abnormalities upon autopsy (53), with this proportion rising to 30% in patients under 40 years of age. These cases without structural abnormalities may result from inherited cardiac channelopathies that disrupt cardiac action potentials. Despite postmortem genetic testing (molecular autopsy), the cause of death remains undetermined in approximately 70% of unexplained SCD cases (46), suggesting the potential role of autoimmune mechanisms in SCD. The incomplete understanding of SCD mechanisms limits the effectiveness of therapeutic interventions. Adami et al.l (54) reported a case of an adult patient with anti-Ro/SSA positive connective tissue disease who developed fatal complete heart block. Although the patient had a normal QTc interval, cardiac arrest ultimately occurred, highlighting that anti-Ro/SSA antibody-associated complete heart block in adults, while potentially insidious, carries a significant risk of fatality. According to a large neonatal cohort study, the incidence of LQTS is approximately 1 in 2,000 individuals, and untreated patients in the highest-risk subgroup have a 10-year mortality rate of up to 50% (55).

### 3.2 Anti-Ro/SSA antibodies and bradyarrhythmias

Research in the last century identified transplacental transfer of anti-Ro/SSA antibodies as a primary cause of congenital AVB (56, 57). Two main theories have been proposed to explain the relationship between anti-Ro/SSA antibodies and CAVB. The inflammation theory posits that anti-Ro/SSA antibodies interact with specific antigens in the conduction tissue, triggering inflammation and fibrosis, which lead to irreversible damage. The electrophysiological theory, by contrast, attributes CAVB to reversible disturbances caused by antibody interference with atrioventricular conduction in experimental models (15). However, neither theory alone fully explains the onset and progression of AVB, leading to the proposal of the calcium channel theory. This theory suggests maternal anti-Ro/SSA antibodies cross-react with calcium channels in fetal cardiomyocytes, initially resulting in an electrophysiological interference on AV conduction, thereby potentially reversible. Prolonged blockade may lead to calcium channel internalization, disrupt intracellular calcium homeostasis, and activate apoptotic and inflammatory mechanisms, ultimately resulting in irreversible fibrotic degeneration of the conduction system and fetal congenital complete AVB (58).The mortality rate for such fetuses *in utero* or during early postnatal life is nearly 30% (59). A similar mechanism can be observed in adults. Subtle structural damage to the fetal conduction system caused by anti-Ro/SSA antibodies may be clinically silent at birth but can progressively worsen with age through mechanisms independent of autoantibodies, manifesting as accelerated age-related degeneration. Consequently, patients may progress from early reversible low-grade AVB (first- or second-degree AVB) to late irreversible third-degree AVB, indicating that late-stage AVB is a progressive disease (34). A retrospective study in Sweden found that approximately 24.5% of patients with unexplained third-degree AVB had mothers who were anti-Ro/SSA antibody-positive, suggesting that congenital conduction abnormalities may go undiagnosed and progress to severe AVB in adulthood (60). These findings indicate that in some adult AVB cases, the etiology stems from unrecognized and untreated CAVB, which progresses and ultimately manifests as severe AVB in adulthood.

Recent national registry data from Denmark indicate that the annual incidence of severe AVB in individuals under 50 years of age is approximately 20 cases per million residents. However, only half of these cases have a clearly identifiable etiology^35^. Anti-Ro/SSA antibodies are not only implicated in CAVB through transplacental transmission (7, 61, 62) but also associated with unexplained AVB in adults (34). Lazzerini et al. (63) proposed three potential types of anti-Ro/SSA antibody-associated AVB in adults (1): acquired type, typically caused by antibody-mediated immune interference and reversible with immunosuppressive therapy (2); late progressive congenital type, associated with structural damage during the fetal period and impossible to reverse with immunotherapy; and (3) mixed type, which may originate from subclinical fetal injury and is compounded by immune-mediated secondary damage in adulthood. In the acquired type, the patients themselves must test positive for anti-Ro/SSA antibodies; in the late progressive congenital type, the mothers must test positive for the antibodies; and in the mixed type, both the patients and their mothers test positive for anti-Ro/SSA antibodies. The study also detailed three cases of different types of AVB, demonstrating the multifaceted impact of anti-Ro/SSA antibodies on the adult cardiac conduction system. With advancing research, anti-Ro/SSA antibodies have been recognized as a major pathogenic factor affecting the adult conduction system. Their mechanisms primarily involve impairing Cav1.2 function while also promoting fibrosis through the induction of apoptosis and inflammation, ultimately leading to conduction system block (34, 64). autoimmune cardiac channelopathies are increasingly recognized as a novel mechanism underlying arrhythmias.

Cav1.2, a voltage-gated calcium channel, plays a critical role in cardiac rhythm and conduction and is widely distributed in contractile cardiomyocytes as well as cells of the atrioventricular and sinoatrial nodes. These channels are composed of multiple subunits, including α1C, α2δ, β, and γ, which together form a complex responsible for calcium ion flux. The α1C subunit constitutes the pore-forming region of the channel, while the α2δ subunit significantly influences its voltage dependence, pharmacological properties, and cell surface expression. Studies have found that serum from anti-Ro/SSA-positive patients exhibits higher binding affinity to five peptides located on the extracellular loops of the S5 and S6 segments (domains I and III) of the Cav1.2 channel (5). Anti-Ro/SSA antibodies can cross-react with the S5-S6 region of the α1C subunit, leading to downregulation of Cav1.2 expression. When Ro/SSA proteins are exposed on the cell surface and recognized by the immune system, further synthesis of autoantibodies accelerates apoptosis (5, 32). Similarly, Maguy et al. (51) demonstrated that purified anti-Cav1.2 IgG from patients with idiopathic cardiac arrest reduces APD by inhibiting calcium channels, thereby inducing arrhythmias. Anti-Cav1.2 antibodies targeting the DIIIE3 domain significantly decreased APD in hiPSC-CMCs (human induced pluripotent stem cell-derived cardiomyocytes) and caused depolarization of the maximum diastolic potential.

Inflammation can directly damage cardiac tissue, potentially leading to atrioventricular conduction delays. Systemic inflammation, mediated by specific cytokines—particularly IL-6—affects the expression of Connexin43, a gap junction protein that connects cardiac cells, enabling the exchange of small molecules and ions and facilitating the rapid propagation of electrophysiological signals. Under inflammatory conditions, elevated IL-6 levels are associated with reduced Connexin43 expression, increasing the risk of atrioventricular nodal conduction delays and block. In guinea pig models, direct administration of IL-6 has been shown to increase the atrioventricular conduction index. *In vitro* experiments further demonstrate that IL-6 significantly reduces Connexin43 protein expression in both cardiomyocytes and macrophages (65).

In addition, Lazzerini et al (4) investigated the prevalence of AVB and the role of anti-Ro/SSA antibodies in young athletes. Among 2,536 athletes, 10 cases of second-degree AVB (II°AVB) were identified, with 30% of these athletes (3/10) testing positive for anti-Ro/SSA antibodies. Notably, the severity of II°AVB was greater in anti-Ro/SSA-positive athletes compared to the negative group. *In vitro* experiments demonstrated that IgG from anti-Ro/SSA-positive patients acutely inhibited ICaL in tSA201 cells and chronically downregulated Cav1.2 expression, altering cardiac electrophysiological properties and leading to AVB. A study on racehorses and mice undergoing high-intensity training showed that even under complete autonomic blockade, a significant reduction in Cav1.2 expression and ICaL density was associated with prolonged PR intervals (66).

A retrospective observational study conducted by Hua et al (67), based on data from Beijing Anzhen Hospital between 2018 and 2022, included 766 patients with heart failure. Among these, 70 patients (9.1%) tested positive for anti-Ro/SSA antibodies, a prevalence higher than that observed in the general healthy population. The results showed that the incidence of AVB and bundle branch block (BBB) was significantly higher in anti-Ro/SSA antibody-positive patients. Even after accounting for known risk factors, including history of autoimmune diseases, anti-Ro/SSA antibodies remained independently associated with AVB and BBB. Recent studies have indicated that the presence of anti-SSA antibodies may be linked to conduction block even in patients without apparent autoimmune diseases (50, 67). Therefore, it is clinically recommended to conduct comprehensive screening, including anti-Ro/SSA antibody testing, in patients with unexplained AVB, even in the absence of clinical signs of autoimmune diseases.

### 3.3 Anti-Ro/SSA antibodies and supraventricular tachyarrhythmias

Anti-Ro/SSA antibody positivity is also associated with increased risk of tachycardia. A large cross-sectional study of 17,231 anti-Ro/La antibody-positive patients and 84,368 controls found higher incidences of paroxysmal supraventricular tachycardia (1.2% vs. 0.9%, P < 0.05) and atrial fibrillation or flutter (7.0% vs. 4.6%, P < 0.001) in the antibody-positive group (50). While these data suggest that anti-Ro/SSA-positive patients have a higher risk of developing supraventricular tachycardia, the specific mechanisms are not yet fully understood.

The binding of anti-Ro/SSA antibodies might trigger inflammatory responses and damage to cardiac conduction tissue, leading to fibrosis. Such damage and fibrosis could disrupt the normal electrical conduction pathways of the heart, thereby increasing the risk of arrhythmias, including supraventricular tachycardia. Multiple studies have linked C-reactive protein and inflammatory cytokines, particularly TNF-α, interleukin-1, interleukin-2, and interleukin-6, to the onset and outcomes of AF (68). Inflammation-induced cardiac channelopathies may predispose individuals to AF in the absence of significant structural heart defects. TNF-α and interleukin-1 increase susceptibility to AF by affecting sarcoplasmic reticulum calcium-handling proteins, such as RyR2 (ryanodine receptor 2), or their associated proteins, thereby enhancing spontaneous diastolic calcium leakage from the sarcoplasmic reticulum (69). Research has shown that TNF-α can induce spontaneous calcium release in murine atrial cardiomyocytes through the reactive oxygen species (ROS) pathway, leading to impaired calcium handling, characterized by reduced calcium transient amplitude and prolonged decay time (70). Furthermore, TNF-α promotes mitochondrial ROS production, enhancing the phosphorylation of calcium/calmodulin-dependent protein kinase II (CaMKII) and RyR2, thereby further driving the development of AF (71).

## 4 Treatment strategies for anti-Ro/SSA antibody-associated arrhythmias

The identification of autoimmune mechanisms provides new therapeutic opportunities for anti-Ro/SSA antibody-associated arrhythmias. Autoantibodies may cause reversible ion channel dysfunction through electrophysiological interference. Therefore, aggressive immunotherapies, such as immunomodulatory drugs, plasma exchange, and immunoadsorption, hold promise for reducing the risk of arrhythmias. These treatments aim to reduce the immune system’s impact on cardiac electrophysiology by targeting pathogenic antibodies. The treatment of anti-Ro/SSA antibody-associated arrhythmias requires a comprehensive approach that addresses both the autoimmune and electrophysiological aspects of the disease. Immunosuppressive therapies, antiarrhythmic medications, and device implantation form the cornerstone of management, while emerging therapies offer hope for more targeted and effective treatments in the future. Early diagnosis and individualized treatment plans remain key to improving outcomes in this patient population.

### 4.1 Basic treatment strategies

Currently, symptom-directed strategies are primarily employed to reduce arrhythmia triggers. Common approaches include lifestyle modifications, avoiding competitive sports, and discontinuing medications that may prolong the QT interval. Additionally, beta-adrenergic blockers and left cardiac sympathetic denervation are recommended to lower arrhythmia risk (72). A large-scale study of 2,536 young athletes revealed a 0.1% prevalence of AVB in the general cohort, while male adolescents undergoing high-intensity training exhibited a 20-fold increased risk (2%, P<0.01), suggesting physical exertion may unmask subclinical conduction abnormalities in susceptible individuals (4). Another study demonstrated that the proportion of patients with QTc ≥500 ms decreased from 18% to 12% among those treated with beta-blockers, and regular risk assessments reduced the need for ICD without increasing the incidence of fatal events (73). Immunosuppressive therapy has shown notable improvement in anti-Ro/SSA antibody-associated AVB, regardless of CTD status (74–76). However, current treatment strategies do not address the underlying molecular mechanisms of LQTS, such as ion channel dysfunction, necessitating the exploration of mechanism-driven therapies.

### 4.2 Immunomodulatory therapy

For adults with anti-Ro/SSA antibody-associated acquired or mixed-type AVB, glucocorticoids and other immunosuppressants could improve cardiac conduction through the following potential mechanisms:(i) suppressing anti-Ro/SSA antibody production, with reduction of their circulating levels, (ii) upregulating L-type calcium channel expression in cardiomyocytes, and (iii)inhibiting inflammatory response, thereby facilitating the recovery of cardiac conduction function. In contrast, the efficacy of immunosuppressive therapy in late-onset congenital AVB, which stems from pre-existing structural damage, is often limited (63). Santos-Pardo et al. (74)reported a case of a 26-year-old woman with anti-Ro/SSA antibody positivity who presented with complete AVB and left bundle branch block. After treatment with methylprednisolone (1 mg/kg/day), her atrioventricular conduction normalized, and her antibody test results turned negative after one year of therapy. This case underscores the importance of long-term immunosuppressive therapy and continuous monitoring in achieving sustained benefits. Lazzerini PE et al. (5) conducted a Steroid Test and found that among 13 patients receiving short-term high-dose glucocorticoid therapy (intravenous methylprednisolone 1 mg/kg/day followed by oral prednisone), all 9 anti-Ro/SSA-positive patients showed improvement in AV conduction, conversion from third-degree AV block to normal conduction or first-degree AV block, with response times ranging from hours to 8 days. In contrast, none of the 4 anti-Ro/SSA-negative patients exhibited any improvement. These findings suggest that glucocorticoids can rapidly reverse anti-Ro/SSA antibody-mediated AV block, possibly by upregulating L-type calcium channel expression in cardiomyocytes, thereby counteracting the acute inhibitory effects of the antibodies. Follow-up data revealed that in some patients, long-term immunosuppressive therapy sustained AV conduction improvements for weeks to over 3 years, indicating that prolonged treatment may maintain efficacy by reducing antibody production. Experimental studies have shown that dexamethasone and other corticosteroids significantly upregulate calcium channels in cardiomyocytes, including enhancing the activity of L-type calcium channels (ICaL) (77–79), with these effects manifesting within 12 to 24 hours, thereby offsetting the acute inhibitory effect of Ro/SSA antibodies on the channels.

Based on these findings, immunomodulatory therapy may be considered in anti-Ro/SSA-positive patients as an alternative strategy or a means to delay pacemaker implantation. However, although promising, further studies are needed to establish stronger evidence for its routine clinical use.

### 4.3 Ion channel modulation therapy

Decoy peptides are a class of small molecules that bind to pathological antibodies, preventing their interaction with cardiac ion channels. These decoy peptides can effectively mitigate the negative impact of anti-Ro/SSA antibodies on cardiac electrophysiology, particularly by targeting the binding sites of antibodies to the Cav1.2 channel. By blocking antibody-mediated inhibition of the ICaL channel, these peptides improve atrioventricular conduction efficiency (5).Li et al. (80) discovered that anti-KCNQ1 autoantibodies, by binding to the extracellular pore region of the KCNQ1 potassium channel, increase IKs current and shorten the QT interval in patients with dilated cardiomyopathy. This finding highlights the potential of immunotherapy targeting KCNQ1 for improving cardiac repolarization. Anti-KCNQ1 autoantibodies enhance IKs current density without altering the channel’s kinetic properties or KCNQ1 protein expression, suggesting modulation through mechanisms such as single-channel conductance or protein interactions. Research by Ange Maguy et al. (81) demonstrated that KCNQ1 antibodies can reverse prolonged cardiac repolarization and reduce arrhythmia risk. In a hiPSC-CM model of LQTS type 1, KCNQ1 antibodies compensated for reduced IKr by enhancing IKs current, thereby shortening the action potential and QT interval. At a concentration of 60 mg/ml, KCNQ1 antibodies significantly increased IKs current density. Although cellular studies highlight the therapeutic potential of KCNQ1 antibodies, further clinical validation is needed to confirm their efficacy and safety. Similarly, Fabris et al. (82) induced the production of antibodies that inhibit IKr by immunizing with peptides derived from the hERG channel pore. Their study found that these antibodies slowed ventricular repolarization, suggesting therapeutic potential for short QT syndrome. Although promising, ion channel modulators remain in early research stages, requiring further optimization of therapeutic dosages and clinical validation.

### 4.4 Implantable cardiac devices

Implantable ICDs are a critical treatment modality for preventing sudden death and terminating arrhythmias in patients with LQTS (83). When considering ICD implantation, it is essential to optimize pharmacological therapy, particularly beta-blockers, before proceeding. Individualized risk assessment is crucial for making informed implantation decisions, avoiding unnecessary ICD placement, and improving therapeutic outcomes (84).

In cases of unexplained AVB, pacemaker implantation is often used to prevent SCD (85). However, this approach may lead to adverse events. A controlled trial showed that patients under 50 years with AVB treated with pacemakers faced a three- to fourfold increased risk of composite endpoints—including death, heart failure hospitalization, and ventricular arrhythmias—compared to controls (HR 3.8; 95% CI 2.9–5.1; P < 0.001) (86). Therefore, more physiological pacing modes, such as biventricular pacing, His bundle pacing, or left bundle branch pacing, may help improve outcomes.

## 4 Conclusion and future directions

Anti-Ro/SSA antibodies, commonly present in connective tissue diseases, are increasingly recognized as significant contributors to adult arrhythmias. Evidence suggests these antibodies interfere with cardiac ion channel function, leading to severe arrhythmias such as QT prolongation, sudden cardiac death, and atrioventricular block. Their interaction with L-type calcium and potassium channels highlights the importance of immune mechanisms in arrhythmogenesis, particularly in unexplained arrhythmias and cardiac arrest, where they may serve as an undiagnosed factor. Beyond anti-Ro/SSA antibodies, the role of other autoantibodies (e.g., anti-NaV1.5) in arrhythmogenesis remains to be fully elucidated. Emerging evidence suggests that these autoantibodies may contribute to the pathogenesis of unexplained arrhythmias, with distinct autoantibodies potentially inducing different arrhythmia subtypes. Consequently, the development of simple, high-throughput detection methods is of paramount importance (10). While their role in fetal arrhythmias is well-studied, the mechanisms underlying their impact in adults remain underexplored. Future research should prioritize investigating these interactions and developing targeted therapies to improve diagnosis and management, ultimately enhancing patient outcomes.
